# Beam-steering of dielectric flat lens nanoantenna with elliptical patch based on antenna displacement for optical wireless applications

**DOI:** 10.1038/s41598-023-43149-z

**Published:** 2023-09-25

**Authors:** Fatma E. Helmy, Ibrahim I. Ibrahim, Amany M. Saleh

**Affiliations:** Electronics and Communications Department, Faculty of Engineering, Helwan University, Cairo, 11795 Egypt

**Keywords:** Engineering, Electrical and electronic engineering, Optics and photonics

## Abstract

In this paper, the switched-beam nanoantenna (NA) concept is introduced with a theoretical design of an inhomogeneous dielectric flat lens modelled with different materials to steer and enhance the radiation in a particular direction based on shifting the illuminator element. Firstly, the design of hybrid plasmonic NA is introduced and analyzed considering different silicon patch shapes such as rectangular, circular, hexagonal, and elliptical shapes. The elliptical patch NA achieves a gain of up to 10.7 dBi and a return loss of − 14.41 dB. Then the design of a gradient-index dielectric flat lens with the NA is introduced to improve the antenna performance by increasing the directivity and consequently decreasing the beam-width. Furthermore, the beam-steering capabilities by displacement of the NA according to different feeding points along the X and Y-direction. By using the gradient-index dielectric flat lens, the gain is increased to 18.4 dBi with an improvement in the return loss reach to − 19.15 dB compared with traditional NA. In addition, the beam-steering capabilities were achieved with a range ± 60° ×  ± 55° with acceptable average antenna gain, side-lobe levels, and half power beam-width of 16.5 dBi, − 12.3 dB and 13.6° respectively.

## Introduction

Recently, the demand for high-speed data rates and capacity is increased. However, the limited Radio Frequency (RF) spectrum in addition to the RF interference puts constraints on these demands. Optical wireless communication (OWC) possesses significant advantages of large communication capacity, good security, concentrated energy, and convenient networking. Optical Nano-Antenna (NA) has attracted a great attention due to its exceptional properties which exist in different applications such as photo-detection, nonlinear plasmonic, medicine, and energy-harvesting applications^1–8^. The optical NA is produced as a device that has the ability to convert the free propagating electromagnetic radiation to localized energy, and vice versa. Moreover, the optical NA is produced as one of the most relevant system components^9–11^ due to its advantages of high performance and low loss in optical wireless communication^12^.

Different materials have been used in the optical NA such as dielectric^13–15^, metallic^16,17^, or hybrid materials^11,18–20^. Silicon has gained significant attention as a dielectric material in various applications such as point-to-point communications and solar cells due to its low cost and abundant fabrication process^21^. Although, the dielectric optical NA exhibits grating lobes, in addition, there is bidirectional radiation in the radiation pattern, which will cause interference and waste power. Plasmonic optical NA has a smaller size and is composed of metal with a size of subwavelength^22–28^. Meanwhile, they exhibit good radiation characteristics and enhance gain. A hybrid plasmonic optical NA has been proposed to combine both advantages of high gain and enhanced radiation efficiency but with difficult fabrication. The metallo-dielectric NA can control light scattering efficiently at the subwavelength range. Furthermore, the hybrid element can also achieve both magnetic and electric resonances. Also, the hybrid plasmonic NA’s footprint is less than that of dielectric grating antennas and plasmonic NAs.

The switched-beam arrays introduced multiple fixed beams that can be readily selected individually. Moreover, the implementation is much simpler than the phased-array NAs. The behavior is a single element of the array is selected for each operation mode. The direction of the beam depends on the position of this element which is activated. The feed antenna array is fixed to the lens collimator’s back surface. The lens performs as a virtual passive phase-shifter, directing incident light to a specific location when used in conjunction with antennas resulting in high gain and directivity. Because of these characteristics, the lens is a useful tool for developing optical beamforming systems. In Refs.^29–36^, different types of metamaterial (gradient-index) lenses have been produced. In Ref.^29^, Fresnel zone plate lenses are provided to correct the phase of the feed antenna leading to an inherently narrowband concept at discrete locations. In Refs.^30–33^, Luneburg lenses are introduced as spherical or hemispherical gradient-index lenses that just one plane of beam scanning is approved. In Ref.^34^, a planar dielectric lens is created, and HFSS is used to simulate its performance. When a microstrip patch antenna is used as the feeding antenna, the performances of a plane layer dielectric lens antenna are examined. It is addressed how far the lens is from the feeding antenna. The unparallel between the feeding antenna and the lens toward the lens antenna as well as the deviation from the central axis are explored. It is possible to create a beam-steerable plane dielectric lens antenna. In Ref.^35^, a design based on a switched-beam array antenna concept with an inhomogeneous dielectric flat lens proposed with different materials to steer the radiation in a particular direction for Millimeter-Wave applications to maintain a flat antenna profile that is much thinner than existing traditionally shaped lenses as achieving beam-scanning in both planes and enabling broadband process. In Ref.^36^, low-temperature co-fired ceramics (LTCC) fabrication is presented, and full laboratory testing of innovative dielectric flat lens antennas for future high data throughput 5G wireless communication systems operating in the 60 GHz range. As a result, the design and numerical simulation of inhomogeneous gradient-index dielectric flat lenses in the optical range for wireless optical communication were presented. This lens’s performance regards to maximum achievable gain, beam-scanning ability, bandwidth efficiency, and overall performance. At 1550 nm, beam-steering techniques are demonstrated in Ref.^37^ using an 8 × 8 hybrid plasmonic NA array operating. The beam is guided in the phased array antenna by predicting the proper feeding phases of the 64 elements using a deep neural network (DNN) with or without a lens. In Ref.^38^, reflective meta-lens and five switchable NAs are coupled in an integrated optical system to offer optical beam steering at the conventional telecommunication wavelength of 1550 nm. In Ref.^39^, two planar lenses are created utilizing, respectively, gradient phase GRPH metasurfaces and gradient refractive index (GRIN) metamaterials. Both of the suggested lenses have the ability to controllably alter the horn antenna’s beam direction. The authors first demonstrate a planar GRIN metamaterial lens that can guide the primary beam of a horn antenna from 08 to 108 and has an index distribution between 1 and 2.45. Additionally, they showed a GRPH metasurface planar lens, which has 108 and 308 degrees of beam deflection, respectively. It’s interesting to note that changing the GRPH metasurface’s lattice constant is all it takes to change the antenna beam’s direction. For low-cost multi-beam antennas in the 5G 26-GHz range, a planar dielectric lens has been developed in Ref.^40^. Four input ports on the stacked-patch microstrip lens feeder allow for four independent high-directivity radiation beams. A maximum steering angle of 25 can be achieved, with a simulated gain of more than 18 dB for all scanning angles. The antenna was created using low-cost manufacturing methods. A beam can be effectively deflected in the desired direction using phase-gradient metasurfaces (PGMs). To illustrate the novel approach to multi-beam antenna design, three metasurfaces with various diffraction orders and energy ratios are created and constructed in Ref.^41^. The diffraction efficiencies for the desired channels are very near to 100%. In order to realize independent holographic pictures in two polarization-preserved fields with various propagating distances, a proof-of-concept metasurface is built in Ref.^42^. It is suggested that a polyatomic unit made up of four different chiral meta-atoms placed in a 2 × 2 array can restrict polarization conversion to enable adequate utilization of the two polarization-preserved components. The suggested mechanism offers a theoretical basis for additional modulations and channel expansions for circular polarization and has enormous promise for wireless communications and multifunctional antenna design.

In this paper, we introduce a beam-steering of dielectric flat lens NA with an elliptical patch based on antenna displacement for optical wireless applications at the standard telecommunication wavelength of 1550 nm (193.5 THz). In “Design considerations and simulation methodology” section, the design considerations and simulation methodology of the plasmonic NAs with various patch geometries and the dielectric flat gradient-index lens are presented with a comparison with the previous publish results to make validation. The results are presented and discussed in “Simulated results and discussion” section. Finally, “Conclusion” section summarizes the findings.

## Design considerations and simulation methodology

**Figure 1:**
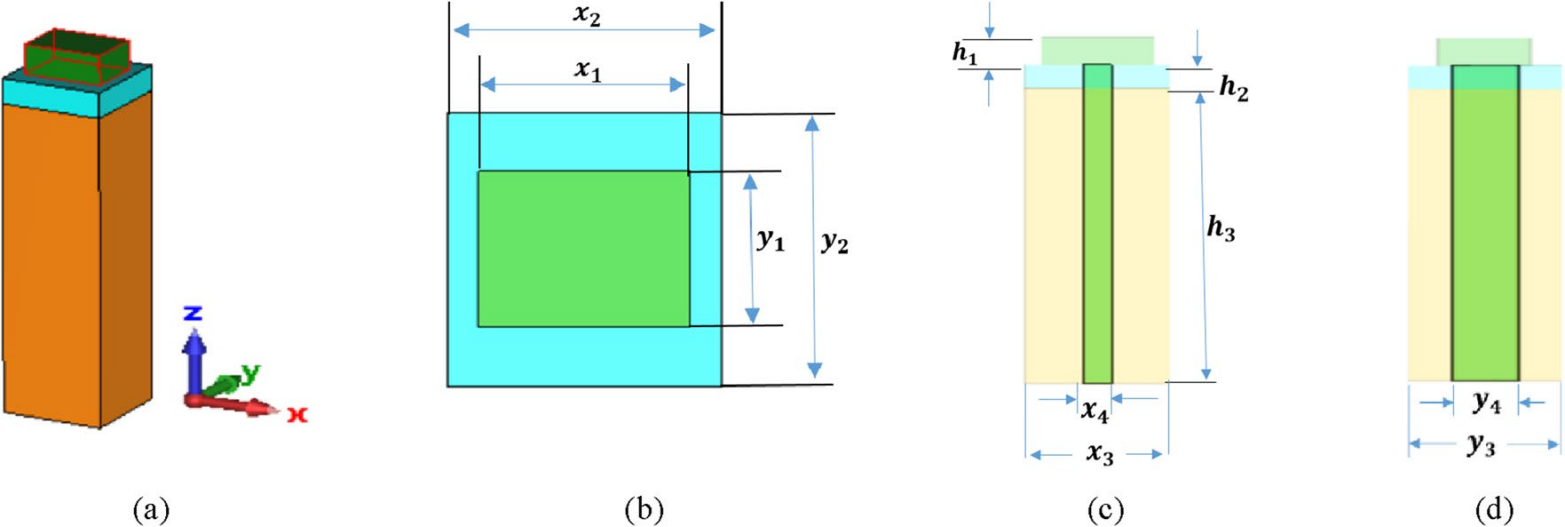
Hybrid plasmonic NA fed by a silicon waveguide in Ref.^26^. (**a**) Perspective view, (**b**) top view, (**c**) front view, and (**d**) side view.

Figure 1 illustrates the schematic diagram of the introduced plasmonic NA in Ref.^26^. The plasmonic NA is composed of a silicon (Si) patch block, a silver (Ag) block, and a silicon waveguide with a silicon dioxide (SiO_2_) coating. The commercial software of Computer Simulation Technology-Microwave Studio (CST-MWS)^43^ is used to investigate the radiation characteristics of the plasmonic NA at the frequency of 193.5 THz (λ = 1550 nm). In order to feed light into the plasmonic NA, it is connected to the silicon patch block via the silicon waveguide that passes through the silver block and is embedded inside the silicon dioxide coating. Light with transverse electric polarization (x polarization) is fed from the bottom of the silicon waveguide and is emitted vertically upward without bidirectional radiation. The lower cutoff frequency for a particular mode in rectangular waveguide is determined by the following equations:

1 $${({f}_{c})}_{mn}=\frac{c}{2}\sqrt{{\left(\frac{m}{a}\right)}^{2}+{\left(\frac{n}{b}\right)}^{2},}$$

where, a, and b denote the width and length of the waveguide. The dominant mode in a particular waveguide is the mode having the lowest cut-off frequency. ‘m’ and ‘n’ represents the possible modes.

The width and length of each block are determined by the length of the edge along the x- and y-axis. The silicon patch block’s width, length, and height are each represented in Fig. 1b,c by the parameters (x_1_, y_1_, and h_1_) are equal to (850, 625, and 300) nm, respectively. Similar to this, the silver block’s width, length, and height are denoted by the parameters (x_2_, y_2_, and h_2_) equal to (1100, 1100, 200) nm, accordingly. Likewise, the parameters (x_3_, y_3_, and h_3_) represent the width, length, and height of the silicon dioxide coating that is equal to (1100, 1100, and 8680) nm, accordingly. The silicon waveguide lengths along the x- and y-axes are denoted by dimensions of (x_4_, y_4_) in Fig. 1c,d which correspondingly equal (220, 450) nm. The relative dielectric constants of the materials of silicon patch and silicon dioxide are 12.11 and 2.1, respectively, with a relative dielectric constant of silver equal to − 129 + j3.28^26^.

### The geometrical structures of the plasmonic NAs

**Figure 2:**
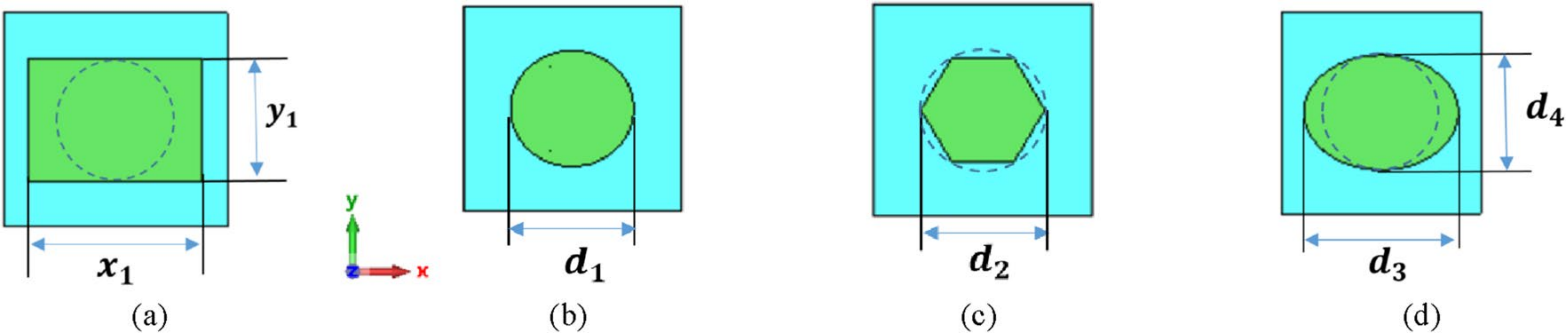
Different patch shapes (**a**) Rectangular^26^, (**b**) Circular, (**c**) hexagonal, and (**d**) elliptical.

The aim of this section is to examine the impact of patch shape on NA characteristics by exploring various shapes including circular, hexagonal, and elliptical. The geometrical structures of different silicon (Si) patch shapes are introduced as shown in Fig. 2. The dimensions of the circular, hexagonal, and elliptical are taken as (d_1_, d_2_, and (d_3_ (major axis), d_4_ (minor axis)) with values of (625, 625, and (850, 625) nm, respectively. The radiation characteristics of the plasmonic NAs produced using CST-MWS and compared with the rectangular patch shape as presented before in Ref.^26^.

### Dielectric flat gradient-index lens design

The operational concept of the dielectric flat lens and its theoretical design are discussed in Refs.^35,36^. The modelled inhomogeneous dielectric flat lens with different materials is used to enhance and steer the radiation in a particular direction. Since the feeding location is adjusted along the x or y-axis, the differing permittivity values provide a linear phase slope, steering the beam only along the gradient-index axis (i.e., along the x or y-axis). The theoretical lens design is composed of six concentric rings of varied permittivity materials (ε_r_) to provide the proper phase delays required for achieving radiation pattern improvement across the lens when lighted from the lens’s focal position. Therefore, the permittivities of the materials in the adjacent rings of the introduced lens had ${\varepsilon }_{{r}_{1}}$ > ${\varepsilon }_{{r}_{2}}$ > ${\varepsilon }_{{r}_{3}}$  > ${\varepsilon }_{{r}_{4}}$> ${\varepsilon }_{{r}_{5}}$ > ${\varepsilon }_{{r}_{6}}$ where the maximum permittivity ${\varepsilon }_{{r}_{1}}$ at the center of the lens and continuous decrease till the minimum permittivity ${\varepsilon }_{{r}_{6}}$ at the outer ring of the lens. Lens design and fabrication with uniform thickness for a flat profile can be determined using Eqs. (2), and (3). The radii (R_i_) for each dielectric zone can be determined by Refs.^29,44^:

2 $${R}_{i}=\sqrt{2Fi\left(\frac{\uplambda }{P}\right)+{\left(i\frac{\uplambda }{P}\right)}^{2}} , \qquad  i=2, 3, \dots, P ,$$

where P is the phase correcting index and is equal to 6, λ is the design wavelength and F is the focal length. The thickness of the lens H is related to the two adjacent permittivities of the lens and it can be obtained by:

3 $$H=\frac{\uplambda }{P(\sqrt{{\varepsilon }_{ri}}-\sqrt{{\varepsilon }_{ri-1}}} , \qquad  i=2, 3, \dots, P .$$

**Figure 3:**
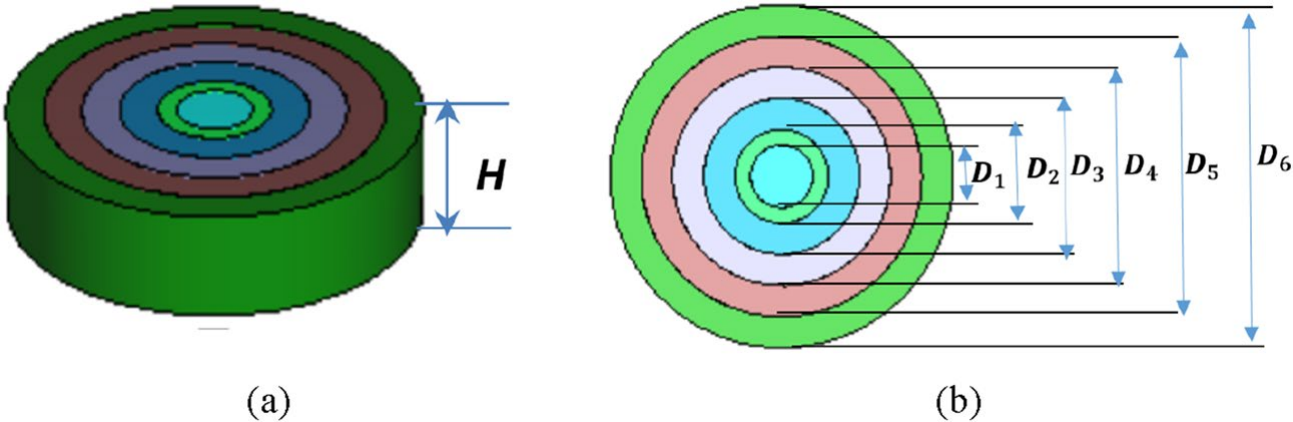
Dielectric flat gradient-index lens structure. (**a**) 3D structure, (**b**) top view.

To achieve the necessary permittivity profile, a Rogers TMM6 dielectric substrate was used^34^, with a maximum permittivity value of 7.1 at the center of lens and a smooth, continuous decrease to 2.9 at the edges (see Fig. 3). The produced dielectric flat gradient-index lens has an outer diameter of lens D_6_ = 7750 nm (5λ)$,$ with a focal length of 1937.5 nm (D/4) and a thickness of 2170 nm (1.4 λ). The outer and inner diameters of the rings are determined by (D_1_, D_2_, D_3_, D_4_, D_5_, D_6_) as seen in Fig. 3b equal to the values (1409, 2113, 3522, 4931, 6340, 7750), respectively. The characteristic parameters of the various dielectric flat lenses are 7.1, 6.79, 6.01, 4.99, 3.92, and 2.9. By adjusting the feeding position in the corresponding direction, the lens’s varied permittivity values provide a phase with a linear slope that steers the beam correspondingly.

## Simulated results and discussion

This section presents and discusses the performance of the introduced hybrid plasmonic NA with varied patch shapes. The radiation pattern characteristic of the dielectric flat lens NA with the elliptical patch is then discussed. Finally, the results of beam-steering by NA displacement will be illustrated.

### The hybrid plasmonic NA with different patch shapes

**Figure 4:**
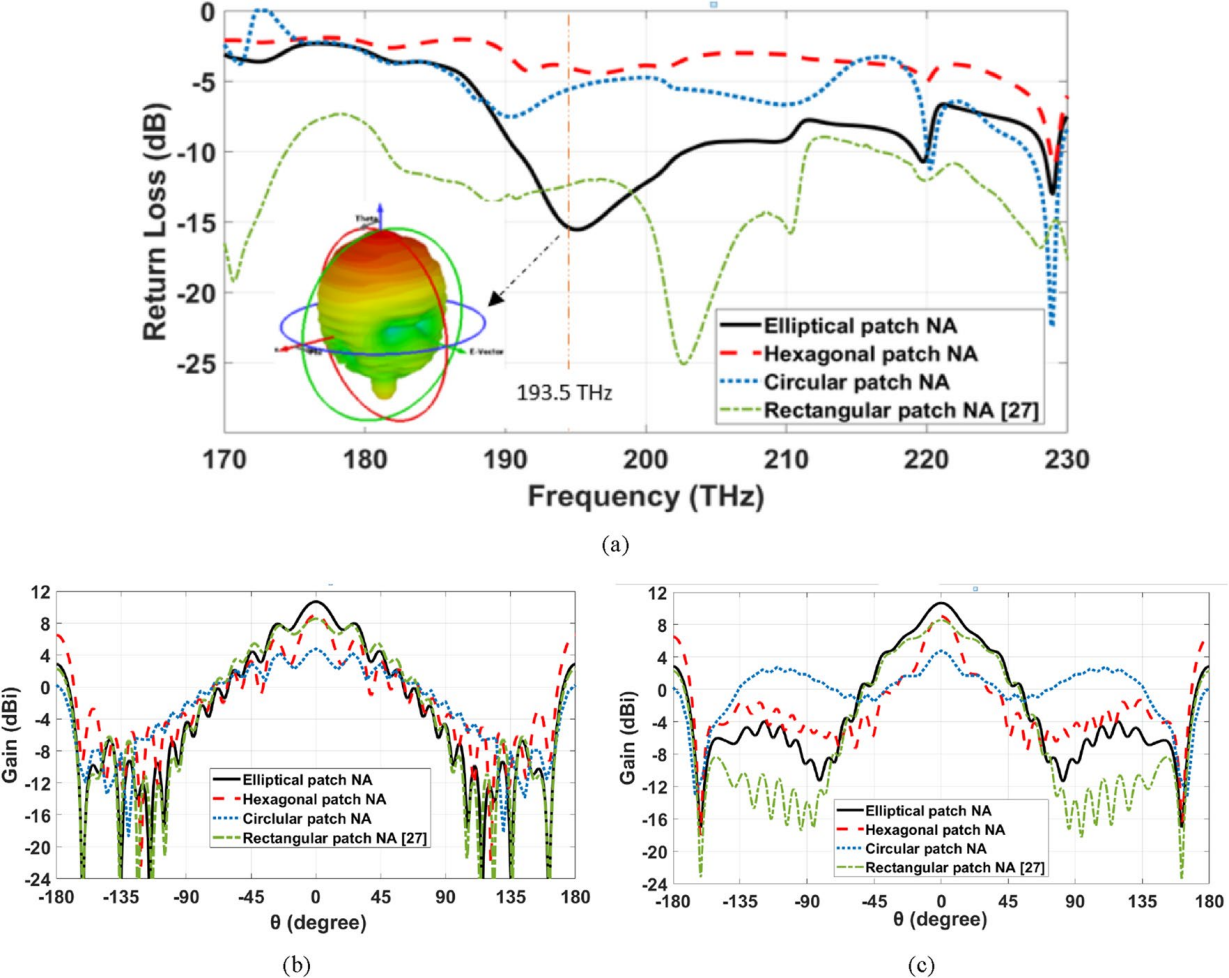
Radiation characteristics of different hybrid plasmonic NAs (**a**) return loss over the frequency range, (**b**) 2-D radiation patterns at 193.5 THz at φ = 0°, (**c**) 2-D radiation patterns at 193.5 THz at φ = 90°.

The return loss over the frequency range of 170 to 230 THz, of the introduced NAs is shown in Fig. 4a. It is noticed that the elliptical patch NA achieves a good matching at the frequency of 193.5 THz compared to other structures. The 3-D radiation pattern of the hybrid plasmonic NA with the elliptical patch shape is inserted inside Fig. 4a, whereas, the elevation and azimuth angles are denoted by θ and φ, respectively. The main lobe of the elliptical patch NA is smooth, and it radiates vertically without bidirectional radiation, as illustrated in the figure with a Side Lobe Level (SLL) of − 2.7 dB. Figure 4b,c show the 2-D radiation patterns of the different hybrid plasmonic NAs at the frequency of 193.5 THz in the x–z plane (φ = 0°) and y–z plane (φ = 90°), respectively. As depicted in Fig. 4b,c, the hexagonal patch NA achieved a gain of 9.02 dBi, which is higher than the rectangular patch NA. This improvement results from the change in the geometrical parameters that more corners are being used^14,15,45^. On the other hand, the radiation pattern characteristic is improved with the elliptical patch NA with a gain reach to 10.7 dBi due to the homogeneous distribution of the supported field intensity compared to 8.4 dBi for rectangular Si-patch NA. Furthermore, the half power beam-width (HPBW) of the proposed elliptical Si-patch NA is found to be 30.2° compared to 64.4° for rectangular Si-patch NA.

**Figure 5:**
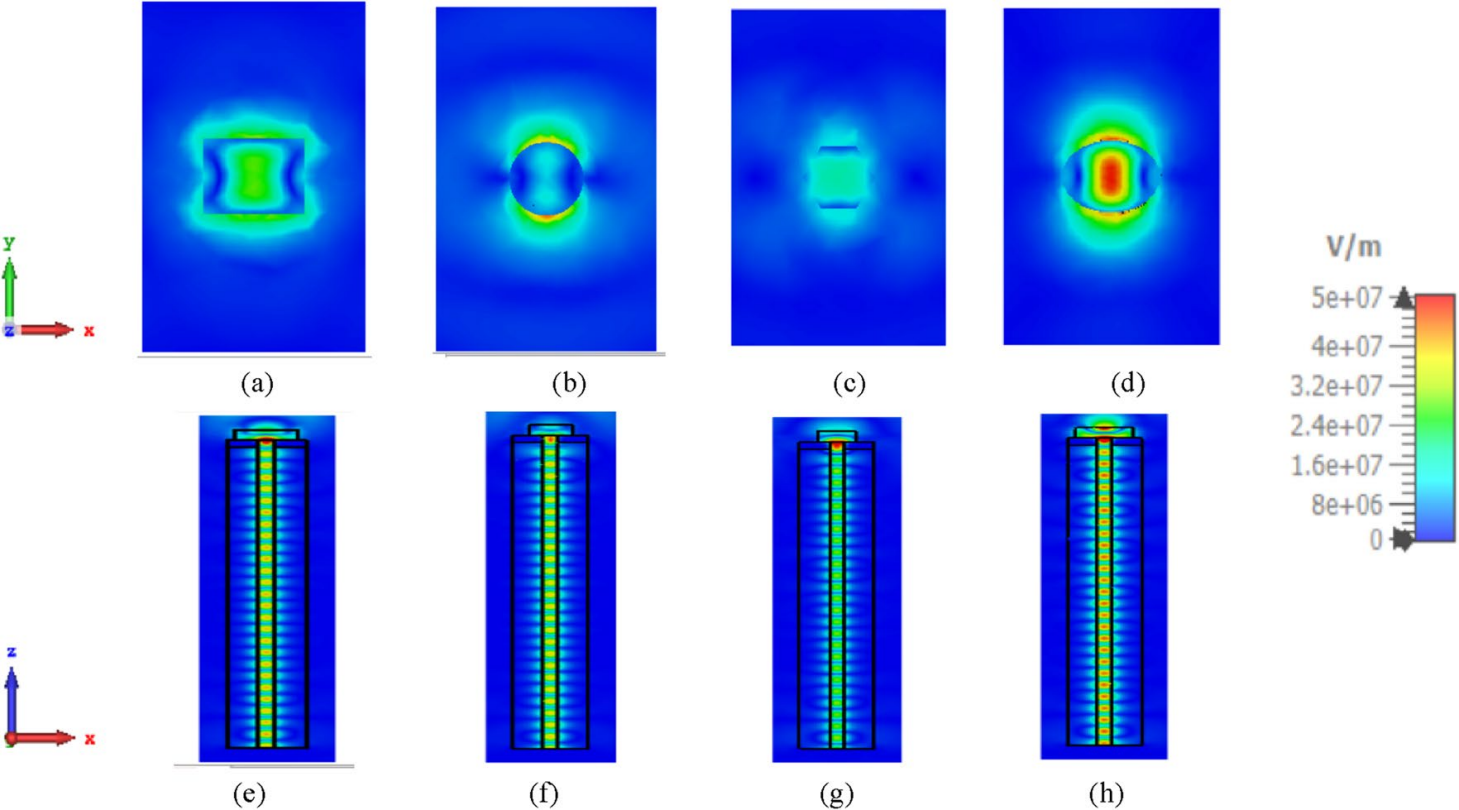
Numerically estimated electric field in the investigated NAs at 1550 nm in x–y, x–z plane. (**a,e**) Rectangular^26^, (**b,f**) circular, (**c,g**) hexagonal, and (**d,h**) elliptical patch NAs.

However, Fig. 5 depicts the transient electric field patterns of the introduced NAs at 1550 nm. The surface plane is indicated above the rectangular^26^, circular, hexagonal, and elliptical patches in Fig. 5a–d, respectively, to show the distributions of electric fields in the x–y plane. Figure 5e–h illustrate the electric field distributions in the x–z plane for rectangular^26^, circular, hexagonal, and elliptical patches, respectively, depicting the travelling wave through a waveguide. It is well known that the localized Surface Plasma Resonance (LSPR) amplifies the electric field at the interface of silver and silicon. From a comparison between the field intensity inside the waveguide in the different patch shapes as in Fig. 5e,f, it is noticed that the intensity improved at the elliptical patch than the rectangular patch due to the improvement in the matching impedance as depicted in the return loss results shown in Fig. 4a. Additionally, the amplified electric field above the patch in elliptical shape NA as shown in Fig. 5d due to the good gain that was obtained. In construction, the electric field distribution of the hexagonal patch NA is seen in Fig. 5c is the worst because of the bad result of return loss shown in Fig. 4a.

To summarize the comparison between different introduced NAs, Table 1 shows the radiation pattern characteristic for the NAs with various Si-patch shapes. It is clear that the antenna’s directivity is primarily determined by its geometrical parameters. Although the rectangular Si-patch NA achieve a total efficiency higher than the elliptical Si-patch NA by 1%, the elliptical patch NA achieved higher gain and better matching with 2.3 dBi and − 1.7 dB, respectively.

**Table 1:** The radiation characteristic comparison for different patch shape NA at 1550 nm.

	Rectangular Si-patch NA^26^	Circular Si-patch NA	Hexagonal Si-patch NA	Elliptical Si-patch NA
Gain (dBi)	8.4	4.79	9.02	**10.7**
Return loss (dB)	− 12.7	− 6	− 3.85	− **14.41**
Radiation efficiency (%)	**95.18**	91.53	81.76	92.4
Total efficiency (%)	**90.03**	68.56	48.03	89.03
SLL (dB)	− **3.1**	− 1.6	− 2.5	− 2.7
HPBW (°)	64.6	66	**22.4**	30.2

### Dielectric flat lens NA with elliptical patch

**Figure 6:**
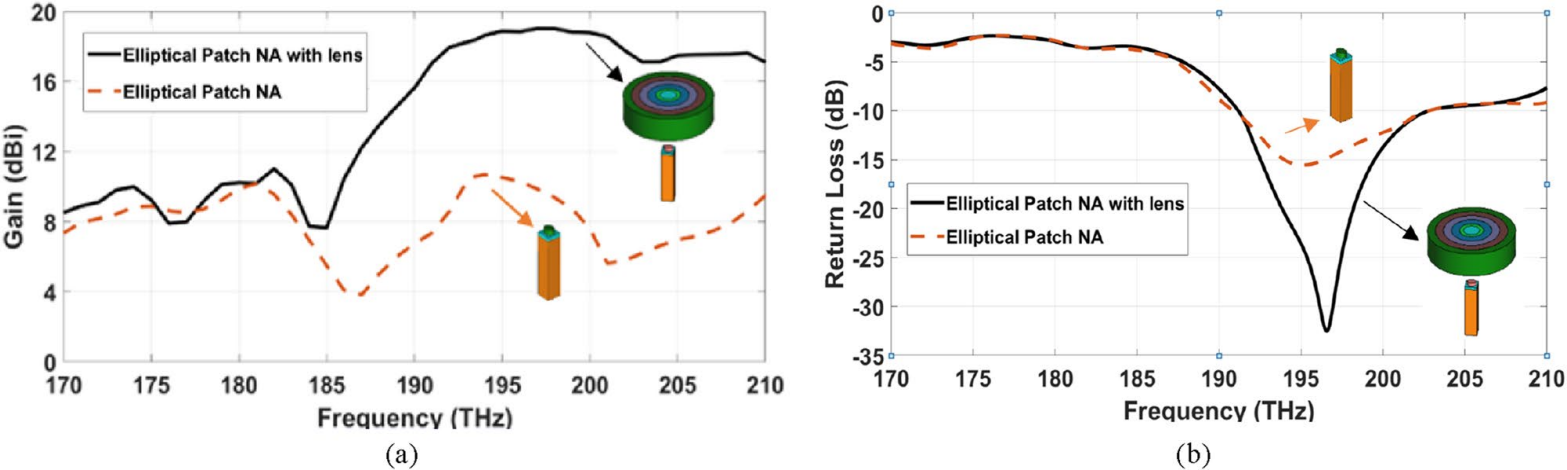
The radiation characteristics comparison of the elliptical NA with lens over the frequency range compared with the investigated NA without lens (**a**) gain, (**b**) return loss.

This section introduces the effect of plate lens antenna on increasing the NA directivity and consequently decreasing the beam-width, whereas, the elliptical plasmonic NA is considered. Figure 6a,b illustrate the gain and return loss of the elliptical NA with lens over the frequency range compared with the investigated NA without lens. As shown in Fig. 6a,b, the achieved gain and return loss of the dielectric flat lens NA with the elliptical patch is enhanced to 18.4 dBi and − 19.15 dB, compared to 10.7 dBi and − 14.41 dB for stand-alone elliptical patch NA without lens, respectively. This improvement is a result of using the low-loss substrate.

**Figure 7:**
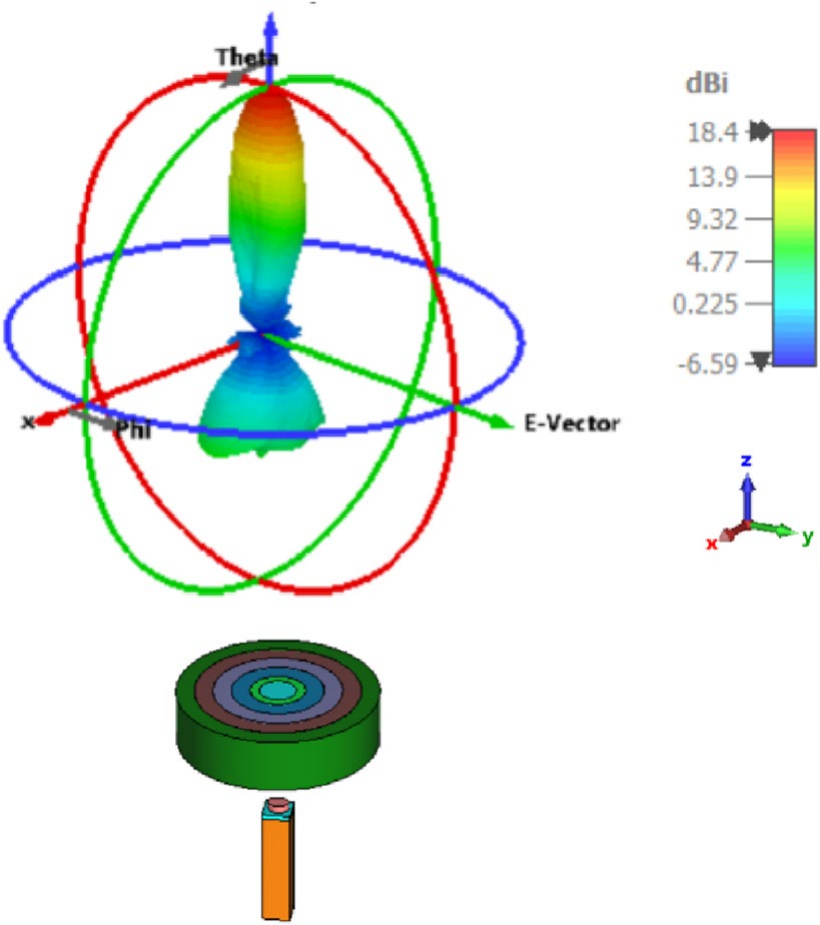
3-D Radiation patterns of dielectric flat lens NA with elliptical patch at 1550 nm.

**Figure 8:**
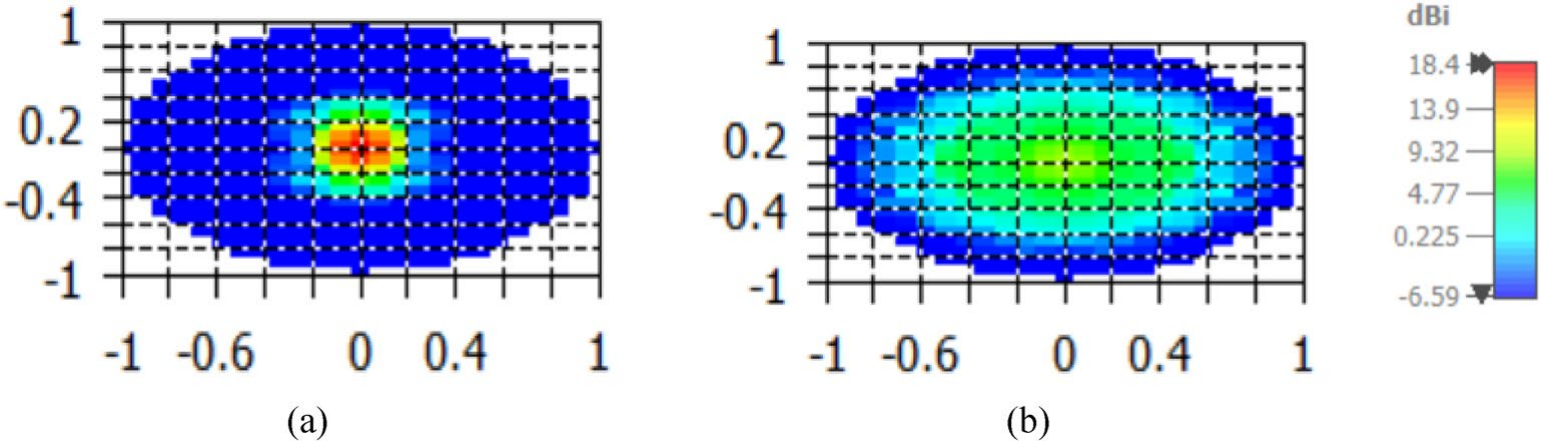
Far-field of the radiation pattern for at 1550 nm for: (**a**) NA with lens, (**b**) NA without lens.

**Figure 9:**
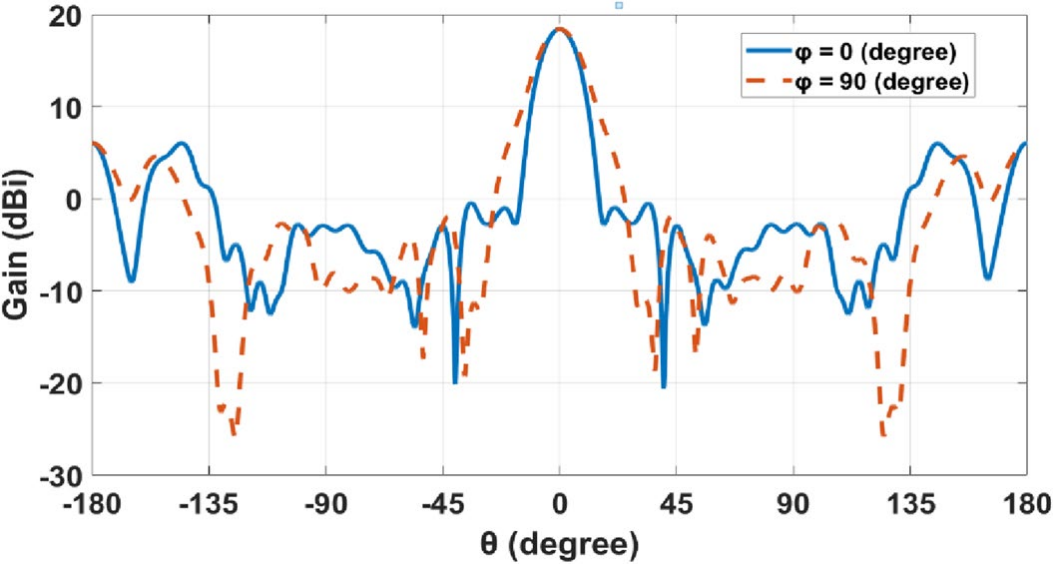
2-D radiation patterns at φ = 0° and φ = 90° planes for dielectric flat lens NA.

The 3D radiation pattern of the elliptical plasmonic NA with the lens at 1550 nm is shown in Fig. 7. It is clear that the major lobe is more directed, but there are also side and back lobes of − 12.3 dB and 6.1 dB, respectively, since there is some back radiation as a result of lens reflections. Moreover, the augmentation of the far-field of the radiation pattern is seen in Fig. 8a of the NA with lens compared to the NA without lens in Fig. 8b. In Fig. 9, 2-D radiation patterns of NA with the lens in the x–z plane (φ = 0°) with the solid blue curve and y–z plane (φ = 90°) with the dashed red curve at a wavelength of 1550 nm. It is found that the HPBW in the x–z plane is 13.6° and has a low SLL of − 12.3 dB.

**Figure 10:**
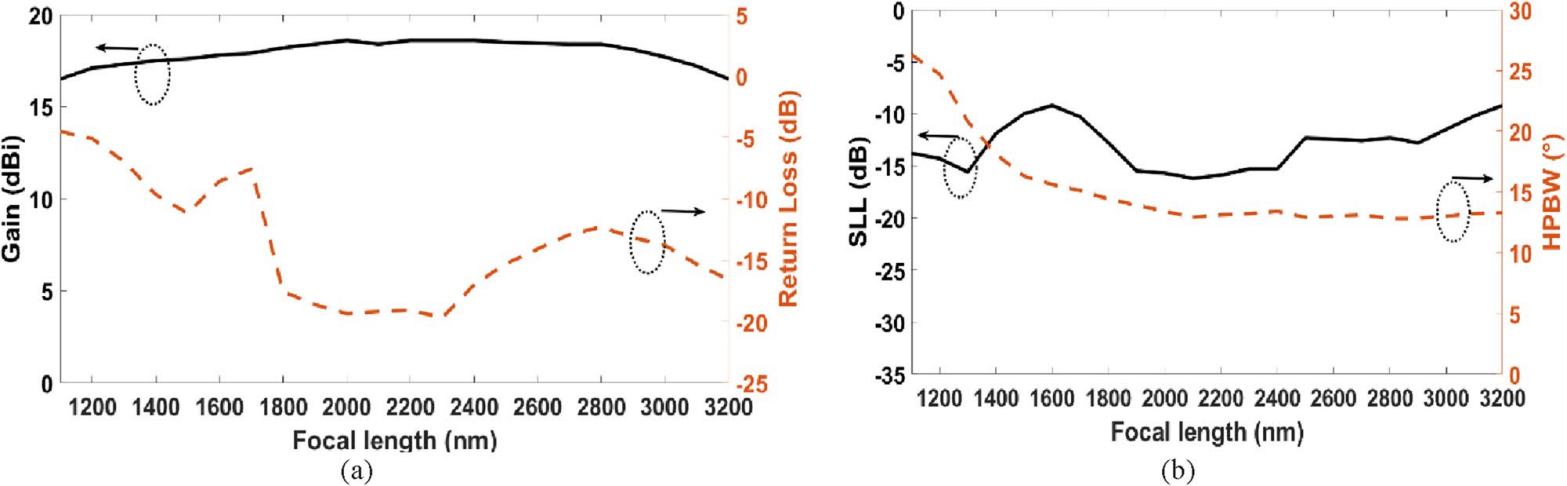
Variation of the focal length versus (**a**) gain and return loss, (**b**) SLL and HPBW.

The focal distance plays a vital role in focusing. The change in return loss, gain, SLL, and HPBW occurs by varying the focal length as shown in Fig. 10. It is shown that the gain does not vary a lot with the change of the focal length from 1100 to 3200 nm with a range between 16 and 18.4 dBi. However, the return loss and SLL improved by changing the focal length in the range from 1850 to 2350 nm with a range between − 17 and − 19.2 dB for the return loss and − 12 dB to − 15 dB. Moreover, HPBW improved from the focal length of 1800 nm to 3200 nm with a range between 15° and 13°.

**Figure 11:**
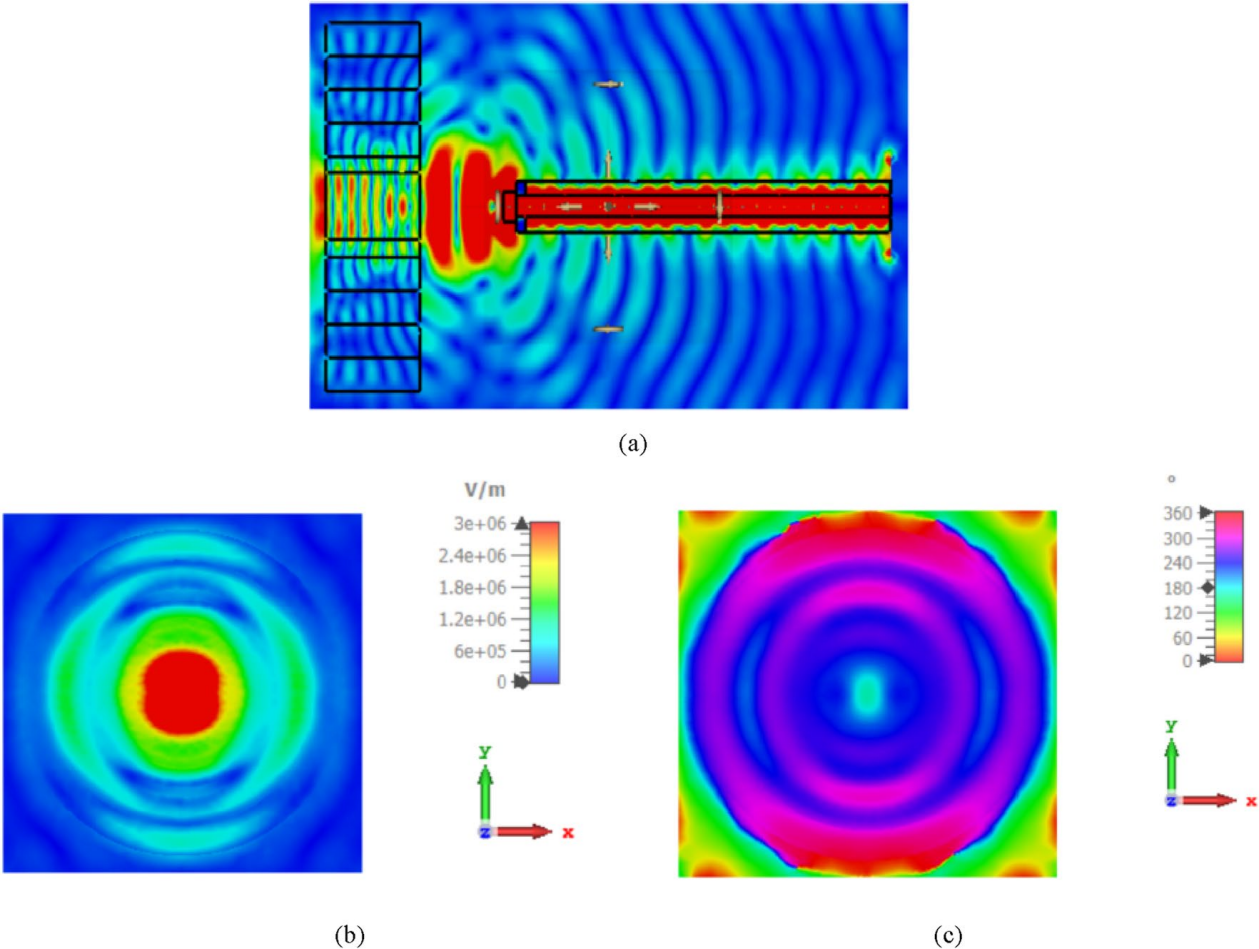
(**a**) Side view of the simulated electric field at 1550 nm in the introduced NA with lens, (**b,c**) simulated amplitude and phase of the electric field at 1550 nm in the aperture of the produced lens, respectively.

Figure 11a depicts the side view of the simulated electric field at 1550 nm in the introduced NA with the lens. However, the amplitude aperture distribution and phase distribution (near field intensity), taken from the surface plane in front of the lens aperture are shown in Fig. 11b,c, respectively. The simulation results illustrate that the produced lens performs well in terms of transforming the incident wavefront into a plane wave while exhibiting low backscatter characteristics.

### Beam-steering based on NA displacement

**Figure 12:**
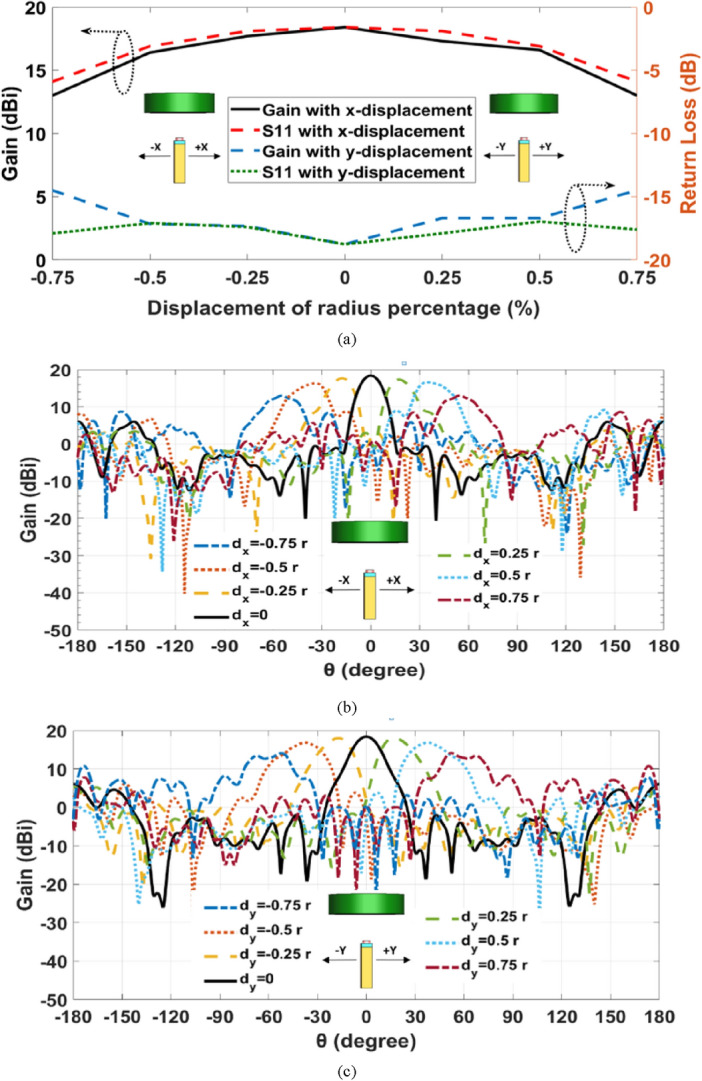
(**a**) The elliptic NA with lens performance with displacement in the X and Y directions at 1550 nm, (**b**) 2-D steering radiation pattern in the x–z plane, (**c**) 2-D steering radiation pattern in the y–z plane.

In this part, the 2-D beam steerable lens antenna with elliptical NA is presented, whereas, beam-steering and switching will be achieved by simply displacing the antenna element. Several displacements of the elliptic patch shape NA have been performed corresponding to various feeding positions along the X-direction and Y-direction. The antenna is displaced in different positions in X and Y directions by d_x_ and d_y_, respectively, related to the radius of the lens (r) as: (0, ± ¼ r, ± ½ r, ± ¾ r). Figure 12a shows the antenna performance of the various positions of the NA displacement in the X and Y directions. It is observed that the beam-steering occurs when the NA position is altered in the x, y-axis. It is seen that the beam shape in the main direction degrades as the placement is increased above ± ¼ r. Also, the SLLs are increased, and HPBWs are lowered for both axes placements. Moreover, a simple improvement in beam-steering with Y displacement in gain and return loss compared with beam-steering with X displacement. The proposed design introduced acceptable beam-steering capabilities with simpler fabrication by one antenna element than the approach of an antenna array. Figure 12b,c show the beam-steering capabilities in the x–z plane (φ = 0°) and y–z plane (φ = 90°), respectively at 193.5 THz by changing the position of the feeding port in the silicon waveguide of elliptical cylinder patch NA in both directions X and Y. It is clear that the beams are differentiated in space when going through the lens producing continuous beam-steering range of about ± 60° ×  ± 55°. In addition to maintaining an acceptable gain value not less than 13 dBi for various steering angles with low side lobe levels of − 4.3 dB and HPBW fluctuating around 18°.

**Figure 13:**
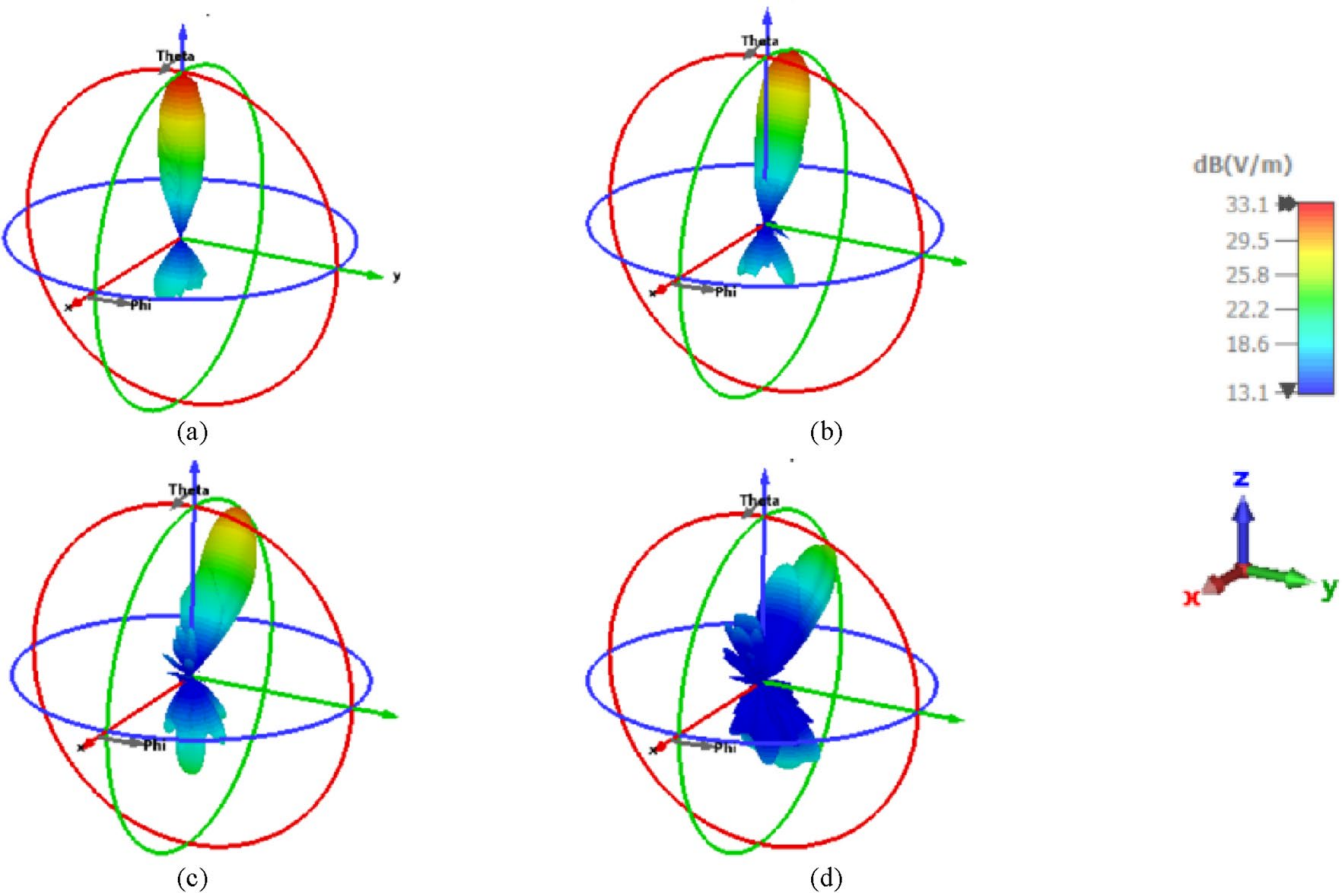
3-D radiation patterns of the elliptical patch shape NA with displacement in the X directions in the negative direction at 1550 nm. (**a**) At the center of the lens, (**b**) ¼ r of the lens, (**c**) ½ r of the lens, and (**d**) ¾ r of the lens.

**Figure 14:**
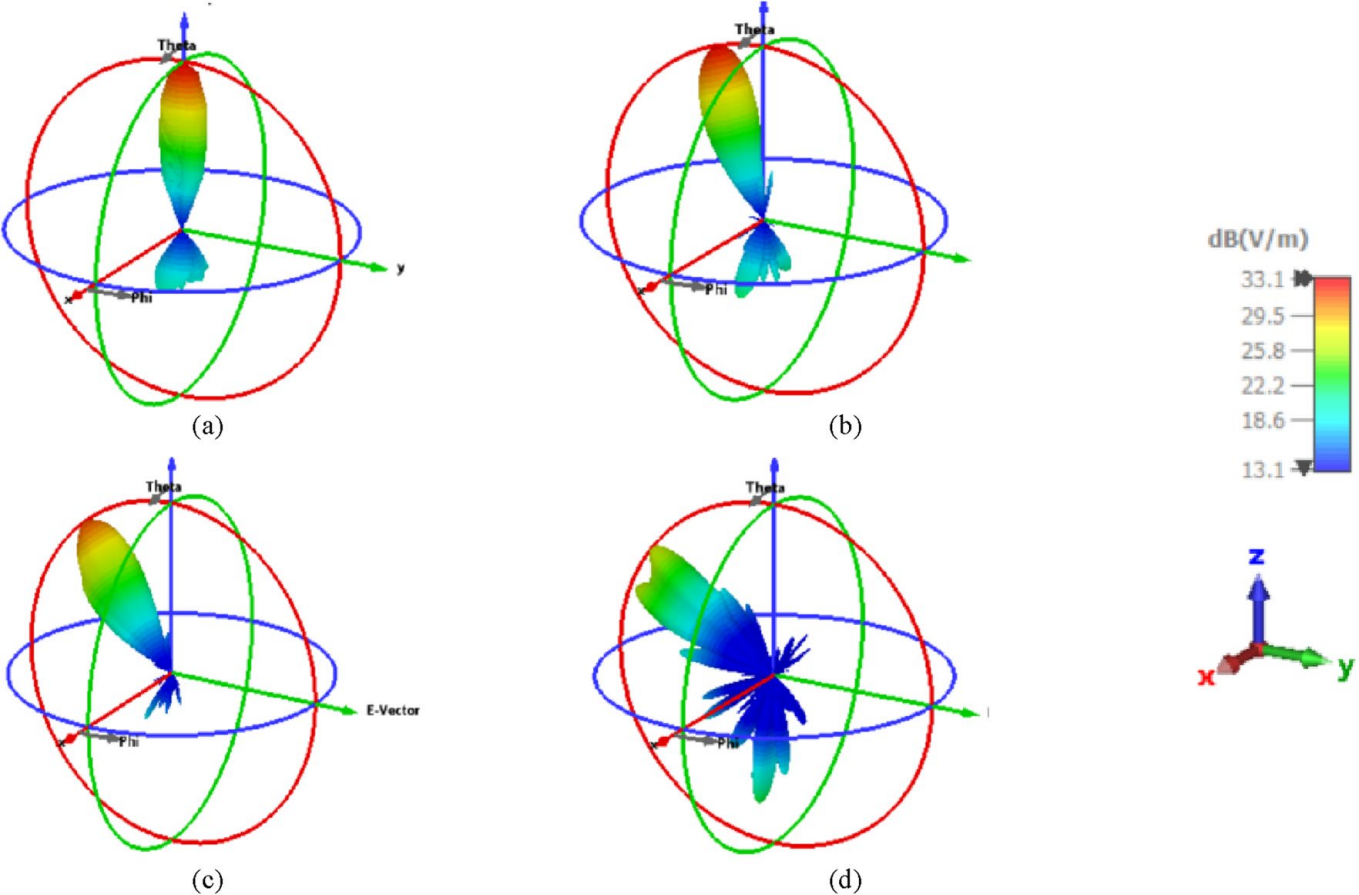
3-D radiation patterns of the elliptical patch shape NA with displacement in the Y directions in the negative direction at 1550 nm. (**a**) At the center of the lens, (**b**) ¼ r of the lens, (**c**) ½ r of the lens, and (**d**) ¾ r of the lens.

Figure 13 illustrates the 3-D radiation patterns of the beam-steering with different positions of the introduced NA with the displacement in the X direction in the negative direction at 1550 nm. In addition, Fig. 14 shows the radiations patterns in 3-D of the NA with displacement in the Y direction in the negative direction. It is shown that beam-steering performance degrades if the position of NA is altered from its initial.

**Figure 15:**
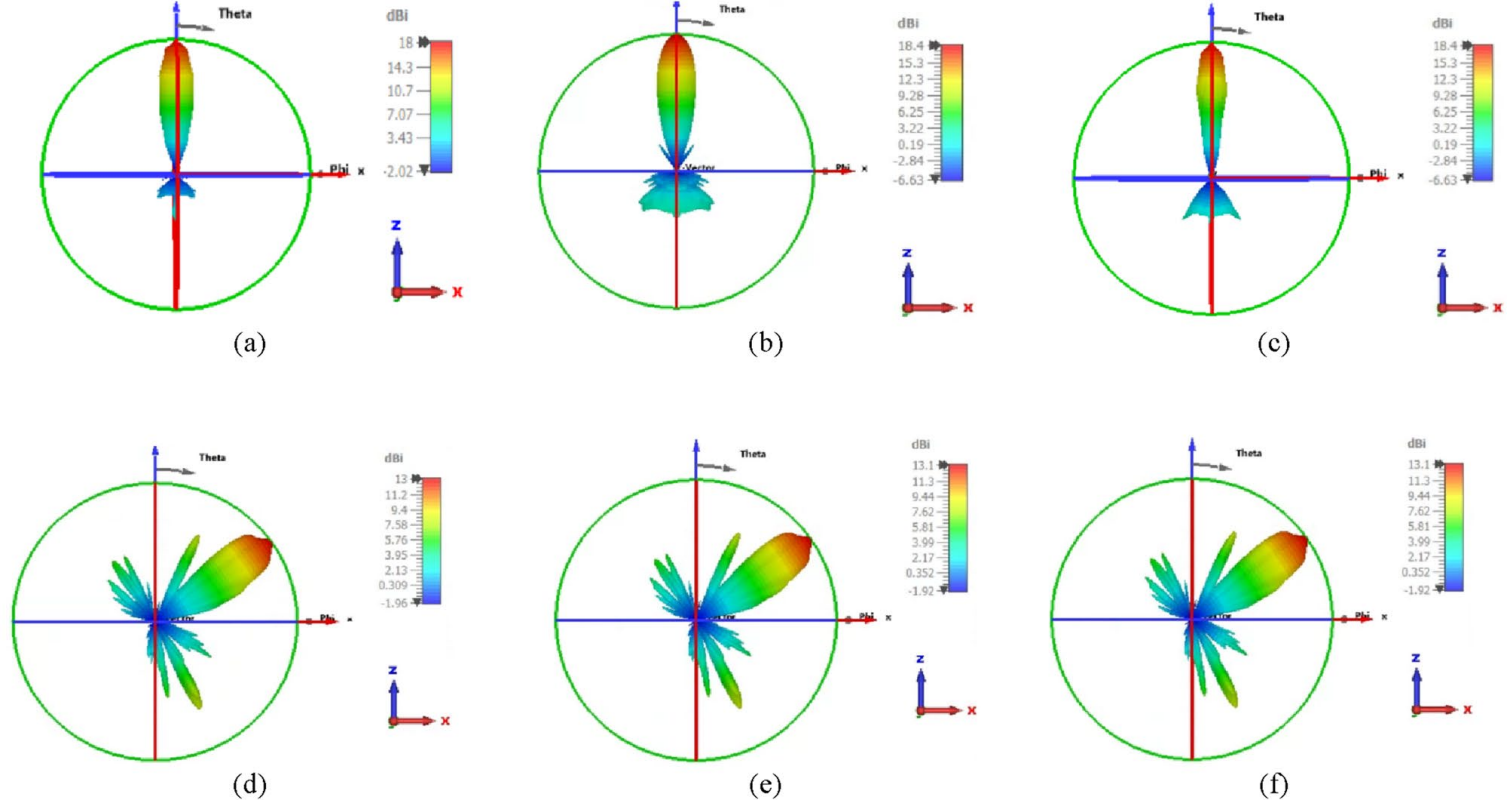
3-D radiation pattern of different focal lengths with (1800 nm, 1937.5 nm, 2200 nm) when the NA’s position: (**a–c**) at focus, (**d–f**) at positions ¾ r of the lens.

However, placing the feed on focus and off focus with different trajectories^46,47^, especially at position ¾ r of the lens was investigated. The used focal length is 1937.5 nm and we consider 1800 nm and 2200 nm to setting up the feeds of the NA. Figure 15a–c illustrates the gain changes with a small various by changing the focal length with 1800 nm, 1937.5 nm, and 2200 nm when the feed sets on focus with values of 18 dBi, 18.4 dBi, and 18.4 dBi, respectively. However, we put the NA at position ¾ r of the lens as a displacement on the x-axis to study the change in focal length with 1800 nm, 1937.5 nm, and 2200 nm as examples in off focus, the gain is considered unchanged with values of 13 dBi, 13.1 dBi, and 13.1 dBi, respectively as shown in Fig. 15d–f. It is evident in Fig. 15a,d that the maximum gain decreased from 18 dBi in on focus to 13 dBi at off focus (position ¾ r) with a focal length of 1800 nm. Additionally, with a focal length of 1937.5 nm, the maximum gain in on focus in Fig. 15b,e drops from 18.4 to 13.1 dBi at off focus. Furthermore, the maximum gain in Fig. 15c,f is altered from 18.4 dBi in on focus to 13.1 dBi at position ¾ r with a focal length of 2200 nm.

## Conclusion

In this paper, the design of hybrid plasmonic NA with different patch shapes such as rectangular, circular, hexagonal, and elliptical shapes operating at 1550 nm are investigated. It is found that the elliptic patch shape NA achieves a higher gain of 10.7 dBi with − 14.41 dB return loss. Then, a dielectric flat lens modelled with different materials is considered to enhance and steer the radiation in a particular direction based on shifting the illuminator element. It is found that the gradient-index dielectric flat lens over elliptic patch shape NA increased the gain to 18.4 dBi, with a return loss of − 19.15 dB. Furthermore, the lens creates the capability to differentiate the beams in space when going through the lens producing a continuous beam-steering range of about ± 60° ×  ± 55° by shifting the NA proportional with various feeding locations along the X and Y-direction with low SLL of − 12.3 dB and HPBW equal to 13.6°.

## Methods

To analyze the performance of the entire structure, a 3D full-wave numerical simulation was performed using CST software and the Uni-directional simulation setup, with the concept design placed into the SiO_2_ background and the boundary conditions defined as open-add-space (modelling the radiation condition). The simulation was run in two stages. The inserted nanoantennas were analyzed using the Finite Elements method during the first stage. Step two involved illuminating the lens with the findings of step one and displacement of the nanoantenna to achieve beam-steering.

## Data Availability

The data sets used and/or analyzed during the current investigation are available upon reasonable request from the corresponding author.

## References

[1] Knight MW, Sobhani HP, Nordlander P, Halas NJ (2011). Photo detection with active optical antennas. Science.

[2] Hussein M, Hameed MFO, Obayya SSA (2017). Nanowires-New İnsights.

[3] Hussein M, Areed NFF, Hameed MFO, Obayya SSA (2014). Design of flower-shaped dipole nanoantenna for energy harvesting. IET Optoelectron..

[4] El-Toukhy YM (2016). Optimized tapered dipole nanoantenna as efficient energy harvester. Opt. Express.

[5] Kauranen M, Zayats AV (2012). Nonlinear plasmonics. Nat. Photon..

[6] Yang Y, Li Q, Qiu M (2016). Broadband nanophotonic wireless links and networks using on-chip integrated plasmonic antennas. Sci. Rep..

[7] El-Toukhy YM (2018). Characterization of asymmetric tapered dipole nanoantenna for energy harvesting application. J. Plasmon..

[8] El-Toukhy YM, Barbillon G (2017). Tapered plasmonic nanoantennas for energy harvesting applications. Nanoplasmonics—Fundamentals and Applications.

[9] Miroshnichenko AE (2011). An arrayed nanoantenna for broadband light emission and detection. Phys. Status Solidi R.

[10] Klemm M (2012). Novel directional nanoantennas for single-emitter sources and wireless nano-links. Int. J. Opt..

[11] Ghanim AM, Hussein M, Hameed MFO, Obayya SSA (2018). Design considerations of super-directive nanoantennas for core-shell nanowires. J. Opt. Soc. Am. B.

[12] Alu A, Engheta N (2010). Wireless at the nanoscale: Optical interconnects using matched nanoantennas. Phys. Rev. Lett..

[13] Krasnok A (2012). All-dielectric optical nanoantenna. Opt. Exp..

[14] Helmy, F. E. *et al*. Optimal design of Yagi-Uda nanoantennas based on elliptical shaped elements. In *Nanophotonics VII (10672)* 106722G (International Society for Optics and Photonics, 2018).

[15] Helmy FE (2019). Effect of Yagi-Uda nano-antenna element shape on the directivity and radiation efficiency. Opt. Quant. Electron..

[16] Taminiau TH, Stefani FD, Hulst NFV (2008). Enhanced directional excitation and emission of single emitters by a nano-optical Yagi-Uda antenna. Opt. Express.

[17] Ma L (2016). Yagi-Uda optical antenna array collimated laser based on surface plasmons. Opt. Commun..

[18] Li Y (2015). Broadband zero-backward and near-zero-forward scattering by metallodielectric core-shell nanoparticles. Sci. Rep..

[19] Meng Z (2017). Ultradirectional optical nanoantennas with high radiation efficiency by core-shell nanoparticles. J. Nanophoton..

[20] Helmy, F. E. *et al*. Metallo-dielectric Yagi-Uda nanoantennas based on rectangular shaped elements. In *Physics and Simulation of Optoelectronic Devices XXVII, SPIE*, Vol. 10912, 203–210 (2019)

[21] Krasnok A (2015). Towards all-dielectric metamaterials and nanophotonics. Metamaterials X.

[22] Wang I, Du Y (2012). Optical input impedance of nanostrip antennas. Opt. Eng..

[23] Iluz, Z. & Boag, A. Wide-angle scanning optical linear phased array. In *IEEE International Conference Microwaves, Communications, Antennas and Electronic Systems* (2015).

[24] Yousefi L, Foster AC (2012). Waveguide-fed optical hybrid plasmonic patch nano-antenna. Opt. Express.

[25] Nia BA, Yousefi L, Shahabadi M (2016). Integrated optical-phased array nanoantenna system using a plasmonic Rotman lens. J. Lightwave Technol..

[26] Xu Y, Dong T, He J, Wan Q (2018). Large scalable and compact hybrid plasmonic nanoantenna array. Opt. Eng..

[27] Alsayed AE, Ghanim AM, Yahia A, Swillam MA (2023). Giant localized electromagnetic field of highly doped silicon plasmonic nanoantennas. Sci. Rep..

[28] Khodadadi M, Nozhat N, Moshiri SMM (2020). Theoretical analysis of a circular hybrid plasmonic waveguide to design a hybrid plasmonic nano-antenna. Sci. Rep..

[29] Petosa A, Ittipiboon A (2003). Design and performance of a perforated dielectric Fresnel lens. IEEE Proc.-Microwaves Antennas Propag..

[30] Peeler G, Coleman H (1958). Microwave stepped-index Luneberg lenses. IRE Trans. Antennas Propag..

[31] Fuchs B (2008). Comparative design and analysis of Luneburg and half Maxwell fish-eye lens antennas. IEEE Trans. Antennas Propag..

[32] Sato K, Ujiie H (2002). A plate Luneberg lens with the permittivity distribution controlled by hole density. Electron. Commun. Jpn..

[33] Zhou B, Yang Y, Li H, Cui TJ (2011). Beam-steering Vivaldi antenna based on partial Luneburg lens constructed with composite materials. J. Appl. Phys..

[34] Zhang, Y., Hong, W. & Zhang, Y. A beam steerable plane dielectric lens antenna. In *2013 Proc. International Symposium on Antennas & Propagation,* Vol. 1, 476–479 (IEEE, 2013).

[35] Imbert M, Papió A, De Flaviis F, Jofre L, Romeu J (2014). Design and performance evaluation of a dielectric flat lens antenna for millimeter-wave applications. IEEE Antennas Wirel. Propag. Lett..

[36] Imbert M (2017). Assessment of LTCC-based dielectric flat lens antennas and switched-beam arrays for future 5G millimeter-wave communication systems. IEEE Trans. Antennas Propag..

[37] Mahmoud KR, Montaser AM (2022). Machine-learning-based beam steering in a hybrid plasmonic nano-antenna array. JOSA B.

[38] Ghaffari V, Yousefi L (2023). Integrated optical beam steering device using switchable nanoantennas and a reflective metalens. Sci. Rep..

[39] Liu Y (2018). Beam steering by using a gradient refractive index metamaterial planar lens and a gradient phase metasurface planar lens. Microw. Opt. Technol. Lett..

[40] Garcia-Marin E (2020). Low-cost lens antenna for 5G multi-beam communication. Microw. Opt. Technol. Lett..

[41] Wang Y (2022). Perfect control of diffraction patterns with phase-gradient metasurfaces. ACS Appl. Mater. Interfaces.

[42] Yuan Y, Wu Q, Burokur SN, Zhang K (2023). Chirality-assisted phase metasurface for circular polarization preservation and independent hologram imaging in microwave region. IEEE Trans. Microw. Theory Tech..

[43] Computer Simulation Technology Microwave Studio (CST MWS). https://www.3ds.com/productsservices/simulia/products/cststudio-suite/ (2022).

[44] Zhang S (2016). Design and fabrication of 3D-printed planar Fresnel zone plate lens. Electron. Lett..

[45] Chao BK (2015). Effects of corner radius on periodic nanoantenna for surface-enhanced Raman spectroscopy. J. Opt..

[46] Hung PV, Dinh NQ, Yamada Y (2022). Negative refractive index-shaped lens antenna with straight line condition for wide angle beam scanning. J. Electromagn. Waves Appl..

[47] Van Hung, P., Dinh, N. Q., Dung, D. T. & Yamada, Y. Caustics and beam steering calculations of negative refractive index lens antenna by the ray tracing method. In *2020 International Conference on Advanced Technologies for Communications (ATC)* 136–139 (IEEE, 2020).

